# Supplementary figures and images for: Genome-wide methylome stability and parental effects in the worldwide distributed Lombardy poplar
Source: BMC Biol. 2024 Feb 5;22:30. doi: 10.1186/s12915-024-01816-1 (PMC10845628; doi:10.1186/s12915-024-01816-1)

## Slide 1
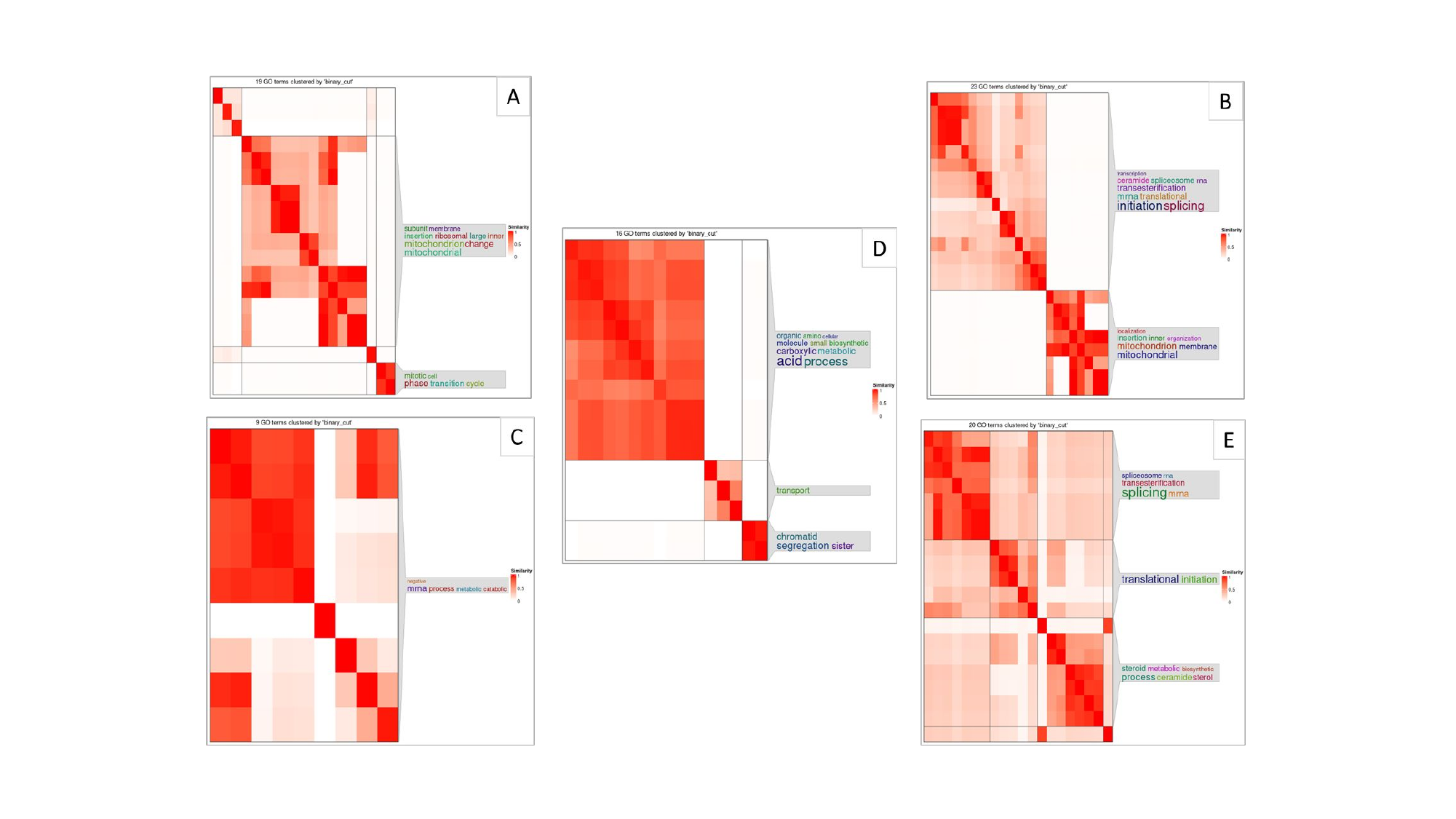

Supplement: Supplementary file 4 — Additional file 4. POWERPOINT-file. Heatmaps with GO terms over-represented in promoters containing DMRs in CpG-context per between-group pairwise comparison. DMRs were identified between groups by grouping the WGBS data from 16 individual Lombardy poplar ramets by their corresponding parent-of-origin (ortet ‘HUN4’ located in Hungary, ‘ITS3’ in Italy, ‘SPC1’ in Spain and ‘UKD2’ in the UK, respectively). A. HUN4 versus ITS3; B. HUN4 versus UKD2, C; ITS3 versus SPC1; D. HUN4 versus SCP1, E. SPC1 versus UKD2. [file 12915_2024_1816_MOESM4_ESM.pptx]
