# Supplementary material for: Genome-wide methylome stability and parental effects in the worldwide distributed Lombardy poplar
Source: BMC Biol. 2024 Feb 5;22:30. doi: 10.1186/s12915-024-01816-1 (PMC10845628; doi:10.1186/s12915-024-01816-1)
Supplement: Supplementary file 6 — Additional file 6. HTML-file with the R source codes to reproduce the results of the bud set analysis. [file 12915_2024_1816_MOESM6_ESM.html]

Bud set model


# Bud set model

#### Pieter Verschelde & An Vanden Broeck

#### 31-08-2022

# 1 Content

This is a condensed R script containing the most important code for importing the data and creating the statistical model. Only the code leading to output in the paper is retained, all code to check all variables and correlations is not included, but all necessary data is bundled with the script.

This document is an R markdown document. The document can be compiled to a html or pdf using knitr (to interpret the R code), and pandoc (to convert this to a layoutted format like html), but is still human readable. The code is placed within chunks and can be run in the R command line. Both knitr and pandoc are bundled with Rstudio, a very popular R editor.

The R packages used are:

```
library(lme4) #mixed effects models
library(mgcv) #general additive models (smoothers)
library(glmmTMB) #alternative way to estimate mixed effect models
library(tidyverse) #toolbox voor coding R using the tidyverse environment
library(sf) #spatial functions
library(stringi) #string operations
library(leaflet) #leaflet graphics
library(simplevis) #wrapper around leaflet graphics to make coding easier
library(ggcorrplot) #make correlation plots of the data
library(ggeffects) #summarise model effects
library(leaflet) #interactive maps
load("my_work_space.RData")
```

# 2 Data

## 2.1 Description

The **Lombardy poplar** is a cultivated clone of *Populus nigra* L. This clonal variety likely originated between 1700 and 1720 from one single male mutant tree located in Asia. The Lombardy poplar can be very easily propagated by cuttings and is distributed worldwide, resulted in several populations of genetically identical trees that have been grown for centuries under local environmental conditions. Its clonal origin in combination with its widespread distribution in space and time, makes the Lombardy poplar and excellent study system to investigate transgenerational environmental and epigenetic effects Vanden Broeck et al. 2018.

Here, we investigate transgenerational phenotypic plasticity (parental effects) in terms of the timing of growth cessation (timing of bud set). The timing of bud set is one cornerstone of the seasonal growth which is strongly adapted to the local climate and known to be under strong genetic control Rohde et al. 2011. The latitude of origin is often strongly correlated with the timing of bud set.

We address the following question: does the ortets’ environment have persistent carry-over effects on the ramets’ phenotype in terms of timing of bud set in a novel environment?

The dataset consists of the bud set data of **Lombardy poplar** ramets (*Populus nigra* cv. *Italica* Duroi) collected from 67 **Lombardy poplar** ortets located over Europe and grown in a novel, common environment near Geraardsbergen (Belgium) (lat. 50,77635°, lon. 3.881007°). We re-analyse the bud set data of (Vanden Broeck et al. 2018) and combine the data with new bud set observations resulting in total data of four consecutive growing seasons. Bud set of the apical bud of the ramets in the common environment experiment was scored during four consecutive growing seasons (2017-2020), using a seven stage scoring system developed for *Populus nigra* by Rohde et al.(2011).

Datasets contains these columns:

1. Basic variables
   - Score: bud set score ( 3 - 2.5 - 2 - 1.5 - 1 - 0.5 - 0) of the ramet. Score three meaning that the apical shoot is fully growing and score zero meaning that the apical bud is formed
   - datum: date of bud set observation (“2017/08/02” - “2020/09/21”)
   - Year: Year of the measurement (2017-2020)
   - DOY: Day of the measurement (183-291)
   - YearDOI: (“2017\_214” - “2020\_265”) so from the 214th day of 2017
   - Location: city of origin of the ortets (28 locations)
   - ID\_TREE: ID of each individual ortet (67 ortets)
   - ID\_cut: ID of each individual ramet (793 ramets)
2. Variables fixed for each ramet during the experiment
   - Weight\_g: (1.2-41.4) Weight(gram) at the beginning before planting
   - F1H2019: (2-58 with 20 NA) shoot growth in 2019 in centimeters
   - F1H2020: (0-92 with 98 NA) shoot growth in 2020 in centimeters
   - MM: (7.0 - 10.0) diameter(in millimeters) of the ramet before planting
   - CN: (8.484 - 16.304 with 966 NA) Initial carbon / nitrogen proportion indicating the nutrient condition of the ortet (Vanden Broeck et al. 2018)
3. Geographic variables and climate data of the ortets’ location. The climate data are obtained from climate data-set CRU TS 2.0; monthly data on a 0.5° x 0.5° grid covering land surfaces (except Antarctica)Harris et al. 2020
   - Lat: Latitude in degrees (40.75-55.89)
   - Lon: Longitude in degrees (-4.593 - 25.457)
   - Elev: Elevation in meter (-1 - 320)
   - JAN: Average temperature in January (°C) for the period 1965 -1994 (-1.4 - 8.0242)
   - JUN: Average temperature in June (°C) for the period 1965 -1994 (13.4 - 20.66)
   - PRATE: Average precipitation (in millimeters) for the period 1965 -1994 (23.27 - 101.07)
   - FRST: Average number of frost days for the period (1965 -1994)(8.497 - 26.436)
4. Climate variables changing over time at the location of the common environment experiment (data from the nearby weather station Hoeilaart)
   - Tmin: (3.382 - 19.302) Minimum temperature (°C) on the observation day
   - Tmax: (14.51 - 39.28) Maximum temperature (°C) on the observation day
   - Tgem: (11.74 - 27.81) Average temperature (°C) on the observation day
   - Tsgem: cumulative average temperature during the experiment (42.78 - 1805.74) (unit: daydegrees) making it an energy variable
   - Tsmin: cumulative minimum temperature during the experiment (26.11 - 1255.16) (unit: daydegrees)

Not all of these variables will be retained for the analysis because of correlation and lesser relevance.

## 2.2 Import data

The data is provided in the file `bud_set_data.csv`

```
#Import the dataset and center some modeling variables
df_raw <- read_csv2("bud_set_data.csv")
str(df_raw)
```

```
## spec_tbl_df [35,950 × 25] (S3: spec_tbl_df/tbl_df/tbl/data.frame)
##  $ Score   : num [1:35950] 2 1 2 2 2.5 2 1 2 1 1.5 ...
##  $ Year    : num [1:35950] 2017 2017 2017 2017 2017 ...
##  $ DOY     : num [1:35950] 214 214 214 214 214 214 214 214 214 214 ...
##  $ ID_cut  : chr [1:35950] "GEH3001" "GEH3002" "GEH3005" "GEH3006" ...
##  $ ID_TREE : chr [1:35950] "GEH3" "GEH3" "GEH3" "GEH3" ...
##  $ Weight_g: num [1:35950] 3.4 2 8.4 2.1 5.1 8.8 3.2 2.7 2.2 3.5 ...
##  $ F1H2019 : num [1:35950] 29 36 38 22 29 18 32 41 4 35 ...
##  $ F1H2020 : num [1:35950] 64 31 56 47 53 31 52 56 16 58 ...
##  $ Location: chr [1:35950] "Duisburg" "Duisburg" "Duisburg" "Duisburg" ...
##  $ Lat     : num [1:35950] 51.4 51.4 51.4 51.4 51.4 ...
##  $ Lon     : num [1:35950] 6.68 6.68 6.68 6.68 6.68 ...
##  $ Elev    : num [1:35950] 30 30 30 30 30 30 30 30 30 30 ...
##  $ JAN     : num [1:35950] 1.9 1.9 1.9 1.9 1.9 1.9 1.9 1.9 1.9 1.9 ...
##  $ JUN     : num [1:35950] 16.5 16.5 16.5 16.5 16.5 ...
##  $ PRATE   : num [1:35950] 69 69 69 69 69 69 69 69 69 69 ...
##  $ FRST    : num [1:35950] 15.5 15.5 15.5 15.5 15.5 ...
##  $ CN      : num [1:35950] 11.5 11.5 11.5 11.5 11.5 ...
##  $ YearDOY : chr [1:35950] "2017_214" "2017_214" "2017_214" "2017_214" ...
##  $ MM      : num [1:35950] 8 8 8 8 8 8 8 8 8 8 ...
##  $ datum   : chr [1:35950] "2017/08/02 00:00:00" "2017/08/02 00:00:00" "2017/08/02 00:00:00" "2017/08/02 00:00:00" ...
##  $ Tmin    : num [1:35950] 13.5 13.5 13.5 13.5 13.5 ...
##  $ Tmax    : num [1:35950] 22.1 22.1 22.1 22.1 22.1 ...
##  $ Tgem    : num [1:35950] 17.3 17.3 17.3 17.3 17.3 ...
##  $ Tsgem   : num [1:35950] 588 588 588 588 588 ...
##  $ Tsmin   : num [1:35950] 405 405 405 405 405 ...
##  - attr(*, "spec")=
##   .. cols(
##   ..   Score = col_double(),
##   ..   Year = col_double(),
##   ..   DOY = col_double(),
##   ..   ID_cut = col_character(),
##   ..   ID_TREE = col_character(),
##   ..   Weight_g = col_double(),
##   ..   F1H2019 = col_double(),
##   ..   F1H2020 = col_double(),
##   ..   Location = col_character(),
##   ..   Lat = col_double(),
##   ..   Lon = col_double(),
##   ..   Elev = col_double(),
##   ..   JAN = col_double(),
##   ..   JUN = col_double(),
##   ..   PRATE = col_double(),
##   ..   FRST = col_double(),
##   ..   CN = col_double(),
##   ..   YearDOY = col_character(),
##   ..   MM = col_double(),
##   ..   datum = col_character(),
##   ..   Tmin = col_double(),
##   ..   Tmax = col_double(),
##   ..   Tgem = col_double(),
##   ..   Tsgem = col_double(),
##   ..   Tsmin = col_double()
##   .. )
##  - attr(*, "problems")=<externalptr>
```

```
df_location <- df_raw %>% 
  distinct(Lat, Lon, Elev, Location) %>% 
  mutate(Location = stri_enc_toascii(Location)) %>% 
  st_as_sf(coords = c("Lon", "Lat"), crs = 4326)

#interactive map with locations of the ortets
leaflet(df_location) %>% 
  addTiles() %>%  
  addCircleMarkers(label = ~Location, color = ~factor(Elev))
```

```
#data subset with the variables of the ramets 
df_cvar <- df_raw %>% distinct(ID_cut, ID_TREE, Weight_g, F1H2019, F1H2020, MM, CN)
summary(df_cvar)
```

```
##     ID_cut            ID_TREE             Weight_g        F1H2019     
##  Length:3162        Length:3162        Min.   : 1.20   Min.   : 2.00  
##  Class :character   Class :character   1st Qu.: 2.80   1st Qu.:20.00  
##  Mode  :character   Mode  :character   Median : 4.40   Median :29.00  
##                                        Mean   : 6.84   Mean   :28.12  
##                                        3rd Qu.: 8.80   3rd Qu.:36.00  
##                                        Max.   :41.40   Max.   :58.00  
##                                                        NA's   :3      
##     F1H2020            CN               MM        
##  Min.   : 0.00   Min.   : 8.484   Min.   : 7.000  
##  1st Qu.:37.00   1st Qu.:11.492   1st Qu.: 7.250  
##  Median :45.00   Median :12.896   Median : 8.000  
##  Mean   :44.93   Mean   :12.763   Mean   : 8.497  
##  3rd Qu.:54.00   3rd Qu.:13.705   3rd Qu.: 9.000  
##  Max.   :92.00   Max.   :16.304   Max.   :10.000  
##  NA's   :16      NA's   :87
```

```
ggcorrplot(cor(df_cvar %>% 
                 select(Weight_g, CN, MM) %>% 
                 na.omit()), 
           type = "lower", lab = TRUE)
```

```
#data subset with the variables of the ortets, containing location and climate data
df_lvar <- df_raw %>% distinct(Location, Elev, Lat, Lon, JAN, JUN, PRATE, FRST)
summary(df_lvar)
```

```
##    Location              Lat             Lon              Elev      
##  Length:39          Min.   :40.75   Min.   :-4.593   Min.   : -1.0  
##  Class :character   1st Qu.:45.93   1st Qu.: 3.696   1st Qu.: 24.5  
##  Mode  :character   Median :47.78   Median : 6.680   Median : 86.0  
##                     Mean   :48.21   Mean   : 9.146   Mean   :113.7  
##                     3rd Qu.:51.09   3rd Qu.:14.703   3rd Qu.:198.0  
##                     Max.   :55.89   Max.   :25.457   Max.   :320.0  
##       JAN               JUN            PRATE             FRST       
##  Min.   :-1.4000   Min.   :13.40   Min.   : 23.27   Min.   : 8.497  
##  1st Qu.:-0.1849   1st Qu.:15.56   1st Qu.: 52.20   1st Qu.:13.368  
##  Median : 2.1000   Median :16.90   Median : 62.00   Median :15.439  
##  Mean   : 2.0127   Mean   :17.24   Mean   : 61.91   Mean   :17.901  
##  3rd Qu.: 3.0510   3rd Qu.:18.70   3rd Qu.: 69.00   3rd Qu.:24.455  
##  Max.   : 8.0242   Max.   :20.66   Max.   :101.07   Max.   :26.436
```

```
#correlation of the climate data at the ortet's location
ggcorrplot(cor(df_lvar %>% select(-Location)), type = "lower", lab = TRUE)
```

```
#data subset of the variables at novel environment, the common environment experiment
df_xvar <- df_raw %>% distinct(Year, DOY, Tsmin, Tsgem, Tmax, Tmin, Tgem)
summary(df_xvar)
```

```
##       Year           DOY             Tmin             Tmax      
##  Min.   :2017   Min.   :183.0   Min.   : 3.382   Min.   :14.51  
##  1st Qu.:2018   1st Qu.:213.2   1st Qu.: 9.255   1st Qu.:19.75  
##  Median :2019   Median :235.0   Median :11.992   Median :22.42  
##  Mean   :2019   Mean   :233.4   Mean   :11.600   Mean   :23.09  
##  3rd Qu.:2019   3rd Qu.:253.5   3rd Qu.:13.341   3rd Qu.:25.35  
##  Max.   :2020   Max.   :291.0   Max.   :19.302   Max.   :39.28  
##       Tgem           Tsgem             Tsmin        
##  Min.   :11.74   Min.   :  42.78   Min.   :  26.11  
##  1st Qu.:15.06   1st Qu.: 590.60   1st Qu.: 405.96  
##  Median :17.11   Median : 966.15   Median : 676.11  
##  Mean   :17.24   Mean   : 921.06   Mean   : 642.71  
##  3rd Qu.:18.55   3rd Qu.:1267.82   3rd Qu.: 900.74  
##  Max.   :27.81   Max.   :1805.74   Max.   :1255.16
```

```
#correlation of the temperature variables at the common environment experiment, DOY and Year
ggcorrplot(cor(df_xvar), type = "lower", lab = TRUE)
```

```
df_xvar %>% ggplot(aes(x = DOY, y = Tsmin, groups = Year)) + 
  geom_point() + geom_smooth() + 
  facet_wrap(~Year)
```

## 2.3 Correlations between explanatory variables

There are many correlations between variables, so a subset of variables is used as basis for the model:

- MM is retained as a characteristic of the ramet because of the lack of correlation and no missing data. It is a proxy of the initial physiological state of the ramet.
- Lat, Lon, Elev, PRATE and Location are retained for the location variables of the ortets, with **focus on Lat as an important variable**. It will function as a proxy for temperature. Location will function as a random effect to explain the location effect not covered with the other variables.
- Tsmin is retained for the energy availability during the common environment experiment. Because it is possible to model DOY on a second degree polynomial of Tsmin with an R2 of .998, DOY contains no extra information compared to Tsmin, and is discarded from the model
- Year is retained as a categorical variable because the contribution of the other variables on the bud set score (response variable) can vary between the years
- Tmax is retained as a temperature variable during the common environment experiment

## 2.4 Aggregating the data of bud set score per ortet

The dataset contains *r nrow(df\_raw)* (35950) records, but the observations are not independent; there are 39 locations and some contain multiple ortets. The in total 67 ortets are represented by multiple ramets (mean: 12.8, range: 4 – 14). These ramets are scored for bud set during several days in four consecutive growing seasons. To simplify the random effects structure of the model, we chose to aggregate the data of bud set score for the different ramets per ortet. This has the advantage that the score for bud set is more finely granulated than the reported steps of 0.5. Models with both the full data and aggregated data where explored but did not result in different interpretations.

In a few cases, we have missing data for the bud set score. When averaging data of the bud set score, ramets with missing data were omitted from data analysis, but this was not influential on the final model.

For the model function, we need a higher than first order polynomial for Tsmin. Therefore, Tsmin is centered and standardized and called Tsminc (the values 600 and 300 are chosen as a round number close to the real mean and sd). The categorical version of the variable Year is called fYear.

```
#aggregated dataset used for modelling

df_agg <-df_work_orig %>% group_by(Year, DOY, YearDOY, ID_TREE, Lat, Lon, Elev, Location, PRATE, Tsmin, Tmax, FRST, MM) %>% 
  summarise(Score = mean(Score), 
            MM = mean(MM),
            ncuts = n()) %>% 
  arrange(Location, ID_TREE, Year, DOY) %>% 
  mutate(fYear = factor(Year),
         Tsminc = (Tsmin - 600)/300, 
         times = factor(YearDOY))

df_agg %>% select(fYear, DOY) %>% 
  group_by(fYear, DOY) %>% 
  summarize(n = n()) %>% 
  mutate() %>% 
  arrange(fYear, DOY)
```

```
## # A tibble: 46 × 3
## # Groups:   fYear [4]
##    fYear   DOY     n
##    <fct> <dbl> <int>
##  1 2017    214    67
##  2 2017    221    67
##  3 2017    228    67
##  4 2017    236    67
##  5 2017    242    67
##  6 2017    250    67
##  7 2017    256    67
##  8 2017    264    67
##  9 2018    183    67
## 10 2018    191    67
## # … with 36 more rows
```

```
str(df_agg, give.attr = FALSE)
```

```
## grouped_df [3,082 × 18] (S3: grouped_df/tbl_df/tbl/data.frame)
##  $ Year    : num [1:3082] 2017 2017 2017 2017 2017 ...
##  $ DOY     : num [1:3082] 214 221 228 236 214 221 214 242 250 256 ...
##  $ YearDOY : chr [1:3082] "2017_214" "2017_221" "2017_228" "2017_236" ...
##  $ ID_TREE : chr [1:3082] "BAZ1" "BAZ1" "BAZ1" "BAZ1" ...
##  $ Lat     : num [1:3082] 44.4 44.4 44.4 44.4 43.2 ...
##  $ Lon     : num [1:3082] 18.04 18.04 18.04 18.04 -4.59 ...
##  $ Elev    : num [1:3082] 233 233 233 233 279 279 0 233 233 233 ...
##  $ Location: chr [1:3082] "\x8eepce" "\x8eepce" "\x8eepce" "\x8eepce" ...
##  $ PRATE   : num [1:3082] 64.6 64.6 64.6 64.6 68 ...
##  $ Tsmin   : num [1:3082] 405 489 574 672 405 ...
##  $ Tmax    : num [1:3082] 22.1 20.9 23.2 22.3 22.1 ...
##  $ FRST    : num [1:3082] 24.1 24.1 24.1 24.1 14.4 ...
##  $ MM      : num [1:3082] 8 8 8 8 8 8 8 8 9 9 ...
##  $ Score   : num [1:3082] 1.318 1.182 0.818 0.5 2.5 ...
##  $ ncuts   : int [1:3082] 11 11 11 11 1 1 14 11 11 11 ...
##  $ fYear   : Factor w/ 4 levels "2017","2018",..: 1 1 1 1 1 1 1 1 1 1 ...
##  $ Tsminc  : num [1:3082] -0.6503 -0.3714 -0.0858 0.2394 -0.6503 ...
##  $ times   : Factor w/ 46 levels "2017_214","2017_221",..: 1 2 3 4 1 2 1 5 6 7 ...
```

## 2.5 Exploratory data analysis

Plots showing the distribution of the bud set scores (response variable) using the aggregated data, i.e. mean score per ortet and per observation day.

```
#distribution of response variable 'bud set score' (without explanatory variables)
#Score three meaning that the apical shoot is fully growing and score zero meaning that the apical bud is formed

ggplot(df_agg, aes(x = Score)) + geom_histogram()
```

```
ggplot(df_agg, aes(x = DOY, y = Score, color = Lat, groups = ID_TREE)) + 
  geom_line() + facet_wrap(~Year)
```

```
ggplot(df_agg, aes(x = Tsminc, y = Score, color = Lat, groups = ID_TREE)) + 
  geom_line() + facet_wrap(~Year)
```

In the first year of the experiment (2017), ramets orignating from ortets growing at lower latitudes (dark blue lines) have slightly higher bud set scores compared to ramets originating from ortets located at higher latitudes (light blue lines), resulting in later bud set and a thus a longer growth period in the novel, common environement for ramets from more southern located ortets in 2017. In the years following 2017, there seem to be no clear differences in the timing of bud set between ramets originating from ortets of different lattitudes.

The data of 2019 can possibly be a bit problematic in the model, because there are many observations with the same score during several observation days. We will fit the model with an autoregressive (AR) model. The autoregressive model specifies that the output variable depends linearly on its own previous values and on a stochastic term (an imperfectly predictable term).

Using Tsminc (cumulative minimum temperature during the experiment) or DOY (Day of the Year) as variable on the x-as does not change the pattern of the bud set.

## 2.6 Model building and selection (Tsmin as quadratic polynomial)

For the model, we have to account for the autocorrelation of bud set scores between ramets originating from the same ortet and for the serial correlation of successive observations of the same ramets in time (during successive days of the year (DOY)). To account for non-independence of the bud set scores of the ramets from a single ortet, we aggregate the data for the ramets per ortet by averaging the bud set scores per ortet. We use a random intercept model, with **random intercepts for the variables Location and ID\_tree**. This accounts for the correlation between ortets (ID\_trees) of a single location. To account for the time series of bud set scores (they can be considered as stationary series), we use **the sample autocorrelation function and an AR(1) model**. An AR(1) model is a linear model that predicts the present value of a time series using the immediately prior value in time.

The full model does not contain `FRST` because of 0.62 correlation with `Lon` and the interaction PRATE:fYear is left out of the full model because of convergence reasons.

The **model selection process** is focused on the likelihood ratio test (LRT; anova in R) based on the p-values between two competing models. When fitting models, it is possible to increase the likelihood by adding parameters, but doing so may result in overfitting. To avoid overfitting, we also used the Bayesian information criterion (BIC) in addition to the LRT, to select the optimal model. The BIC generally penalizes free parameters more strongly than the Akaike information criterion.

The final selected, simplified model (based on LRT and BIC) is significantly worse than the full model based on LRT only. However, the relative contributions of the eliminated variables/predictors on the total prediction of the response variable (bud set score) where small.

We use the R package *glmmTMB* Brooks et al. 2017 that can be used to model zero-inflated count data.

The selected model is:

Score ~ Tsminc + I(Tsminc^2) + Lat + fYear + (1 | Location/ID\_TREE) +  
(ar1(times + 0 | fYear)) + Tsminc:fYear + I(Tsminc^2):fYear + Lat:fYear

```
#full model, interaction PRATE:fYear did not converge,
#so PRATE is in the model without interaction

#no autocorrelation (convergence problems)
modelfull <- glmmTMB(data = df_agg, 
                Score ~ (Tsminc + I(Tsminc^2) +  Lat + MM + Tmax + Lon) * fYear + 
                  PRATE + 
                  (1|Location/ID_TREE))

#model without autocorrelation
modelchosen <- glmmTMB(data = df_agg, 
                Score ~ (Tsminc + I(Tsminc^2) +  Lat) * fYear + 
                  (1|Location/ID_TREE))

#model with autocorrelation
modelchosenac <- update(modelchosen, .~. +  
                  ar1(times + 0|fYear) + 
                  (1|Location/ID_TREE))

anova(modelfull, modelchosen, modelchosenac)
```

```
## Data: df_agg
## Models:
## modelchosen: Score ~ (Tsminc + I(Tsminc^2) + Lat) * fYear + (1 | Location/ID_TREE), zi=~0, disp=~1
## modelchosenac: Score ~ Tsminc + I(Tsminc^2) + Lat + fYear + (1 | Location/ID_TREE) + , zi=~0, disp=~1
## modelchosenac:     (ar1(times + 0 | fYear)) + Tsminc:fYear + I(Tsminc^2):fYear + , zi=~0, disp=~1
## modelchosenac:     Lat:fYear, zi=~0, disp=~1
## modelfull: Score ~ (Tsminc + I(Tsminc^2) + Lat + MM + Tmax + Lon) * fYear + , zi=~0, disp=~1
## modelfull:     PRATE + (1 | Location/ID_TREE), zi=~0, disp=~1
##               Df     AIC     BIC  logLik deviance  Chisq Chi Df Pr(>Chisq)    
## modelchosen   19 1457.47 1572.10 -709.74  1419.47                             
## modelchosenac 21 -628.26 -501.56  335.13  -670.26 2089.7      2     <2e-16 ***
## modelfull     32 1451.44 1644.51 -693.72  1387.44    0.0     11          1    
## ---
## Signif. codes:  0 '***' 0.001 '**' 0.01 '*' 0.05 '.' 0.1 ' ' 1
```

```
summary(modelchosenac)
```

```
##  Family: gaussian  ( identity )
## Formula:          
## Score ~ Tsminc + I(Tsminc^2) + Lat + fYear + (1 | Location/ID_TREE) +  
##     (ar1(times + 0 | fYear)) + Tsminc:fYear + I(Tsminc^2):fYear +  
##     Lat:fYear
## Data: df_agg
## 
##      AIC      BIC   logLik deviance df.resid 
##   -628.3   -501.6    335.1   -670.3     3061 
## 
## Random effects:
## 
## Conditional model:
##  Groups           Name          Variance Std.Dev. Corr      
##  ID_TREE:Location (Intercept)   0.009550 0.09773            
##  Location         (Intercept)   0.001741 0.04172            
##  fYear            times2017_214 0.051073 0.22599  0.68 (ar1)
##  Residual                       0.042081 0.20514            
## Number of obs: 3082, groups:  ID_TREE:Location, 67; Location, 28; fYear, 4
## 
## Dispersion estimate for gaussian family (sigma^2): 0.0421 
## 
## Conditional model:
##                        Estimate Std. Error z value Pr(>|z|)    
## (Intercept)            1.527410   0.303492   5.033 4.83e-07 ***
## Tsminc                -1.021387   0.205483  -4.971 6.67e-07 ***
## I(Tsminc^2)            0.296964   0.239973   1.237 0.215905    
## Lat                   -0.015672   0.004760  -3.292 0.000994 ***
## fYear2018             -0.509005   0.302575  -1.682 0.092521 .  
## fYear2019              0.711582   0.292294   2.434 0.014913 *  
## fYear2020             -0.774865   0.314615  -2.463 0.013782 *  
## Tsminc:fYear2018       0.388809   0.230146   1.689 0.091143 .  
## Tsminc:fYear2019       0.251938   0.219637   1.147 0.251355    
## Tsminc:fYear2020      -0.099743   0.237672  -0.420 0.674730    
## I(Tsminc^2):fYear2018 -0.502256   0.253741  -1.979 0.047770 *  
## I(Tsminc^2):fYear2019 -0.477372   0.246624  -1.936 0.052913 .  
## I(Tsminc^2):fYear2020  0.052193   0.275269   0.190 0.849616    
## Lat:fYear2018          0.020450   0.003093   6.612 3.80e-11 ***
## Lat:fYear2019          0.016168   0.002934   5.510 3.59e-08 ***
## Lat:fYear2020          0.018717   0.003214   5.823 5.78e-09 ***
## ---
## Signif. codes:  0 '***' 0.001 '**' 0.01 '*' 0.05 '.' 0.1 ' ' 1
```

```
# residuals vs. fitted values plot for verifying the assumptions of a linear model (normally distributed errors)
df_agg_augm <- df_agg
df_agg_augm$fitted <- fitted(modelchosenac)
df_agg_augm$residuals <- resid(modelchosenac, type = "p")

# total data
ggplot(df_agg_augm, aes(x = fitted, y = residuals)) + 
  geom_point() + geom_smooth()
```

```
# separate plot per year. in 2019 the model fit increased using autocorrelation compared to the model without autocorrelation
ggplot(df_agg_augm, aes(x = fitted, y = residuals)) + 
  geom_point() + geom_smooth() +
  facet_wrap(~fYear)
```

```
#residuals are distributed more around the center than expected in a normal distribution
ggplot(df_agg_augm, aes(sample = residuals)) + geom_qq() +   geom_qq_line()
```

```
ggplot(df_agg_augm) + geom_histogram(aes(x = residuals))
```

## Model prediction

Prediction of bud set score of the ramets in the novel, common environment experiment in time (Tsmin: cumulative minimum temperature during the experiment) using the selected model.

In 2017; ramets originating from ortets from lower lattitudes show higher bud set scores (longer vegetative growth period), compared to ramets originating from ortets from higher lattitudes, thus from more northern locations. This effect is, however, very small.

```
#dummy location and tree added to df, because expected by predict, but they are not used
#because we predict on the population level
df_preds <- expand.grid(Lat = c(42:54), 
                            fYear = unique(df_agg$fYear), 
                            Tsminc = seq(-2,2,by = 0.1),
                        Location = "dummy_location",
                        ID_TREE = "dummy_tree")


predictions_tsmin <- predict(modelchosen, 
                       newdata = df_preds %>% filter(Lat %in% c(42,46,50,54)), 
                       re.form = ~0, 
                       se.fit = TRUE, 
                       allow.new.levels = TRUE)

predictions_lat<- predict(modelchosen, 
                       newdata = df_preds %>% filter(Tsminc %in% c(1.5, 0.5, -0.5,-1.5)),
                       re.form = ~0, 
                       se.fit = TRUE, 
                       allow.new.levels = TRUE)

predictions_df_tsmin <- as.data.frame(predictions_tsmin) %>% 
  bind_cols(df_preds %>% filter(Lat %in% c(42,46,50,54))) %>% 
  mutate(lcl = qnorm(0.025, mean = fit, sd = se.fit), 
         ucl = qnorm(0.975, mean = fit, sd = se.fit))

predictions_df_lat <- as.data.frame(predictions_lat) %>% 
  bind_cols(df_preds %>% filter(Tsminc %in% c(1.5,0.5, -0.5,-1.5))) %>% 
  mutate(lcl = qnorm(0.025, mean = fit, sd = se.fit), 
         ucl = qnorm(0.975, mean = fit, sd = se.fit))

#with confidence intervals (effect of latitude is very small)
predictions_df_tsmin %>% 
  filter(Tsminc >= -1.5 & Tsminc <= 1.5) %>% 
  ggplot(aes(x = (Tsminc * 300 + 600), y = fit, ymin = lcl, ymax = ucl, 
             color = factor(Lat))) +
  geom_ribbon(alpha = 0.1) +
  geom_line() + 
  facet_wrap( ~ fYear, nrow = 1) + 
  labs(x = "Tsmin", y = "Fitted bud set score", color = "Latitude" )
```

```
#without confidence intervals (score not bounded between 0 and 3)
predictions_df_lat %>% 
  ggplot(aes(x = Lat, y = fit, ymin = lcl, ymax = ucl, 
             color = factor(Tsminc))) +
    geom_ribbon(alpha = 0.1) +
  geom_line() + 
  facet_wrap( ~ fYear)
```

## 2.7 Model

```
model <- gamm(data = df_agg, 
              Score ~ fYear + Lat + Lat:fYear + s(Tsminc, by = fYear),
              random = list(Location= ~ 1, ID_TREE= ~ 1),
              correlation = corCAR1(form = ~ jitter(DOY) | Location/ID_TREE))
AIC(model$lme, modelchosenac)
```

```
##               df       AIC
## model$lme     20 -502.0783
## modelchosenac 21 -628.2593
```

```
qqnorm(resid(model$lme));qqline(resid(model$lme))
```

```
plot(model$lme)
```

```
plot(model$gam)
```

```
summary(model$lme)
```

```
## Linear mixed-effects model fit by maximum likelihood
##   Data: strip.offset(mf) 
##         AIC       BIC   logLik
##   -502.0783 -381.4117 271.0392
## 
## Random effects:
##  Formula: ~Xr - 1 | g
##  Structure: pdIdnot
##              Xr1      Xr2      Xr3      Xr4      Xr5      Xr6      Xr7      Xr8
## StdDev: 2.369423 2.369423 2.369423 2.369423 2.369423 2.369423 2.369423 2.369423
## 
##  Formula: ~Xr.0 - 1 | g.0 %in% g
##  Structure: pdIdnot
##            Xr.01    Xr.02    Xr.03    Xr.04    Xr.05    Xr.06    Xr.07    Xr.08
## StdDev: 1.053316 1.053316 1.053316 1.053316 1.053316 1.053316 1.053316 1.053316
## 
##  Formula: ~Xr.1 - 1 | g.1 %in% g.0 %in% g
##  Structure: pdIdnot
##            Xr.11    Xr.12    Xr.13    Xr.14    Xr.15    Xr.16    Xr.17    Xr.18
## StdDev: 3.219546 3.219546 3.219546 3.219546 3.219546 3.219546 3.219546 3.219546
## 
##  Formula: ~Xr.2 - 1 | g.2 %in% g.1 %in% g.0 %in% g
##  Structure: pdIdnot
##            Xr.21    Xr.22    Xr.23    Xr.24    Xr.25    Xr.26    Xr.27    Xr.28
## StdDev: 1.288722 1.288722 1.288722 1.288722 1.288722 1.288722 1.288722 1.288722
## 
##  Formula: ~1 | Location %in% g.2 %in% g.1 %in% g.0 %in% g
##          (Intercept)
## StdDev: 0.0008269999
## 
##  Formula: ~1 | ID_TREE %in% Location %in% g.2 %in% g.1 %in% g.0 %in% g
##         (Intercept) Residual
## StdDev:   0.1048176 0.211287
## 
## Correlation Structure: Continuous AR(1)
##  Formula: ~jitter(DOY) | g/g.0/g.1/g.2/Location/ID_TREE 
##  Parameter estimate(s):
## Phi 
##   0 
## Fixed effects:  y ~ X - 1 
##                              Value Std.Error   DF   t-value p-value
## X(Intercept)             1.5656048 0.2438524 3005  6.420296  0.0000
## XfYear2018              -0.8958557 0.2010623 3005 -4.455613  0.0000
## XfYear2019               0.3188837 0.1944875 3005  1.639610  0.1012
## XfYear2020              -0.6543663 0.2069938 3005 -3.161284  0.0016
## XLat                    -0.0146651 0.0042603   38 -3.442247  0.0014
## XfYear2018:Lat           0.0204499 0.0031919 3005  6.406878  0.0000
## XfYear2019:Lat           0.0161676 0.0030281 3005  5.339234  0.0000
## XfYear2020:Lat           0.0187169 0.0033171 3005  5.642582  0.0000
## Xs(Tsminc):fYear2017Fx1 -0.7108386 1.0169437 3005 -0.698995  0.4846
## Xs(Tsminc):fYear2018Fx1 -0.2980111 0.2923795 3005 -1.019261  0.3082
## Xs(Tsminc):fYear2019Fx1  0.0885684 0.0983453 3005  0.900586  0.3679
## Xs(Tsminc):fYear2020Fx1 -1.1921090 0.4798209 3005 -2.484487  0.0130
##  Correlation: 
##                         X(Int) XfY2018 XfY2019 XfY2020 XLat   XY2018: XY2019:
## XfYear2018              -0.621                                               
## XfYear2019              -0.642  0.779                                        
## XfYear2020              -0.603  0.732   0.756                                
## XLat                    -0.855  0.349   0.361   0.339                        
## XfYear2018:Lat           0.384 -0.777  -0.482  -0.453  -0.450                
## XfYear2019:Lat           0.405 -0.492  -0.762  -0.477  -0.474  0.632         
## XfYear2020:Lat           0.370 -0.449  -0.464  -0.784  -0.433  0.577   0.609 
## Xs(Tsminc):fYear2017Fx1 -0.200  0.243   0.251   0.236   0.000  0.000   0.000 
## Xs(Tsminc):fYear2018Fx1  0.000  0.053   0.000   0.000   0.000  0.000   0.000 
## Xs(Tsminc):fYear2019Fx1  0.000  0.000   0.001   0.000   0.000  0.000   0.000 
## Xs(Tsminc):fYear2020Fx1  0.000  0.000   0.000  -0.009   0.000  0.000   0.000 
##                         XY2020: X(T):Y2017 X(T):Y2018 X(T):Y2019
## XfYear2018                                                      
## XfYear2019                                                      
## XfYear2020                                                      
## XLat                                                            
## XfYear2018:Lat                                                  
## XfYear2019:Lat                                                  
## XfYear2020:Lat                                                  
## Xs(Tsminc):fYear2017Fx1  0.000                                  
## Xs(Tsminc):fYear2018Fx1  0.000   0.000                          
## Xs(Tsminc):fYear2019Fx1  0.000   0.000      0.000               
## Xs(Tsminc):fYear2020Fx1  0.000   0.000      0.000      0.000    
## 
## Standardized Within-Group Residuals:
##        Min         Q1        Med         Q3        Max 
## -5.5660010 -0.5520342  0.0118445  0.5347133  5.6726505 
## 
## Number of Observations: 3082
## Number of Groups: 
##                                                       g 
##                                                       1 
##                                              g.0 %in% g 
##                                                       1 
##                                     g.1 %in% g.0 %in% g 
##                                                       1 
##                            g.2 %in% g.1 %in% g.0 %in% g 
##                                                       1 
##              Location %in% g.2 %in% g.1 %in% g.0 %in% g 
##                                                      28 
## ID_TREE %in% Location %in% g.2 %in% g.1 %in% g.0 %in% g 
##                                                      67
```

```
# predict(model$lme)

df_preds_tsmin <- ggpredict(model, , terms = c("Tsminc", "Lat", "fYear"))
plot(df_preds_tsmin)
```

```
df_preds_lat <- ggpredict(model, , terms = c("Lat", "Tsminc", "fYear"))
plot(df_preds_lat)
```

```
save.image (file = "my_work_space.RData")
```

**Conclusion**: There is a statistical significant effect of the latitude-of-origin (Lat) on bud set phenology of the Lombardy poplar ramets but the estimated effect is very small and only of biological significance in the first year of the experiment (2017). In 2017, moving ramets northward from the latitude of the donor tree, thus into longer days, resulted in a slightly increase in bud set score and thus a slightly longer growth period for these ramets relative to ramets originating from the latitude nearby the experimental site. Although still statistical significant, the effect size of the latitude-of-origin (Lat) is near zero in the years 2018, 2019 and 2020 and of non-biological relevance.

In 2017 was the effect of the cumulative minimum temperature at the experimental site (Tminc; centered and standardized) on the predicted values for the bud set score about two orders of magnitude larger compared to the effect of the latitude-of-origin (Lat) and thus clearly of more importance.
